# A three-year prospective cohort study evaluating implant stability utilising the Osstell® and Periotest™ devices

**DOI:** 10.3389/fdmed.2023.1139407

**Published:** 2023-03-13

**Authors:** Ian Reynolds, Lewis Winning, Ioannis Polyzois

**Affiliations:** Department of Restorative Dentistry and Periodontology, Dublin Dental University Hospital, Trinity College Dublin, Dublin, Ireland

**Keywords:** implant stability measurement using two devices, osseointegration, implant stability, bone, resonance frequency analysis, damping capacity assessment

## Abstract

**Objectives:**

To investigate implant stability measurements from two different devices and at three different time points in order to determine their level of correlation. To also evaluate the influence of a range of clinical characteristics on the values produced by the devices at these three time points.

**Materials & Methods:**

Measurements were recorded at implant placement (T1), implant exposure (T2) and at 3 years from implant placement (T3). A range of clinical data was collected including patient demographics and site characteristics. Stability measurements and clinical characteristics were recorded for 29 patients and 68 dental implants at T1, subsequent stability measurements were recorded for 67 implants at T2 and 58 implants at T3. Correlation testing between the Osstell® and Periotest™ devices was carried out utilising Spearman's rank correlation for each time point. Analysis of the difference between clinical factors and stability measurements was compared using Kruskal-Wallis test for each variable and time point.

**Results:**

A single dental implant failed shortly after second stage surgery for an overall survival rate of 98% during the study timeline. The median ISQ value was 73.25 (IQR 67–75) at T1 and 74 (IQR 70.5–77) at T3. The median Periotest value was −4 (IQR −6, −2) at T1 and −6 (IQR −7, −5) at T3. The range of ISQ values observed was 50 (39–89) ISQ at T1 and decreased to 21 (61–82) ISQ at T3. The Periotest values ranged from 37 (29 to −8) at T1 and decreased to 6 (−2 to −8) at T3. A weak to moderate correlation was observed between mean ISQ and Periotest values across time points T1, T2 and T3, (*r* = −0.26, *p *= 0.05), (*r* = −0.35, *p *< 0.01) and (*r* = −0.28, *p *= 0.04) respectively.

**Conclusions:**

Based on the results of this study there was a weak to moderate level of correlation between values recorded between the two measurement devices at implant placement, implant exposure and three years following placement. For both the Osstell® and Periotest™ a narrowing of the range of stability values was observed from T1 to T3. In general, Periotest™ seemed to be more sensitive in highlighting differences in measurements affected by local conditions.

## 1. Introduction

Implant stability is fundamental to the concept of osseointegration and can be described as either primary or secondary. Sennerby & Meredith described primary stability as a mechanical phenomenon that develops into secondary stability as part of the osseointegration process. This mechanical mechanism is related to the physiological process of bone remodelling and resorption that occur at the tissue-implant interface (1).

Primary stability is dictated by the stiffness of the object or in clinical terms the rigidity of the dental implant in the osteotomy site. Stiffness is defined as the extent to which the implant resists deformation in response to an applied force. The two main factors that influence primary stability are (1) the mechanical properties of the bone at the site of implant placement and (2) how well the fixture is engaged with the osseous tissue as determined by surgical technique and implant geometry (2). Secondary stability is established gradually as bone resorption and remodelling occur at the implant-tissue interface. The main determinants of secondary stability are primary stability, bone remodelling and implant topography (3, 4). Implant stability has also been proposed as the absence of mobility and defined as the ability to support an axial, lateral or rotational load. Thus, implant stability is now accepted as an integral feature of successful osseo-integration and evaluation of implant stability is an essential component of implant therapy geometry (2).

Despite this reinforcement of osseointegration and implant stability as the keystone feature of successful implantology, the diagnostic methods available to clinicians to objectively evaluate implant stability have failed to progress commensurately with other aspects of research in implant dentistry. The ability to measure implant stability can provide valuable information to support clinical decision-making in implant therapy and improve communication, case documentation and trust between clinicians and patient (5). However, the traditional methods available in practice are invasive, subjective or of limited quantifiable value such as clinical perception, removal torque assessment, and percussion testing of the implant with a blunt instrument (2, 6, 7). To address the limitations of traditional approaches, new quantitative, non-invasive methods and devices have been developed to evaluate implant stability (8). Two such commercially available devices are the Osstell® ISQ which is based on resonance frequency analysis technology and the Periotest™ device based on damping capacity assessment technology. Several studies have identified some degree of association between some clinical characteristics and these quantitative measurement devices (9–11); however, other publications have produced conflicting results (12). It is also important to highlight the limitations of much of the literature with a trend for a strong correlation in laboratory studies and a weaker to no correlation in clinical studies. This may be due to methodological heterogeneity in such studies. In conclusion further robust and well-structured research is essential to clarify this aspect of implant dentistry and enhance our armamentarium in the clinical management of implantology.

The primary aim of this study, therefore, was to investigate the performance of the Osstell® ISQ and the Periotest™ device against each another. Specifically, to determine if there is a correlation between the values produced by the Osstell® ISQ and the Periotest^™^ device at implant insertion, following integration of the fixture and 3 years following placement. Secondly, we investigated if certain clinical or other patient characteristics can affect the values produced by the two devices at these three time-points.

## 2. Materials and methods

### 2.1. Study design

This study was a prospective clinical study that evaluated the stability of dental implants at three different time points employing two different measurement devices. It primarily tested how the output values from the two devices correlated with each other at T1, time of implant placement; T2, time of second stage surgery; and T3, 3 years from implant placement. It also investigated the relationship between the implant stability data and selected clinical characteristics. Ethical approval was granted by the Research Ethics Committee of Dublin Dental University Hospital and the Joint Research Ethics Committee in St. James' Hospital (Reference number 2018-08).

### 2.2. Study population

The sample population was recruited from a consecutive group of patients referred to the periodontal department of the Dublin Dental University Hospital for provision of dental implants. Participants were either referred from their general practitioner or from another department in the Dublin Dental University Hospital. Inclusion criteria included all male or female patients aged 18 years or older requiring at least one dental implant.

Exclusion criteria comprised participants that required substantial bone grafting procedures (such as block grafting, ridge expansion procedures, vertical augmentations) prior to potential implant placement; acute/chronic auto-immune mucosal diseases (i.e., pemphigus, lichen planus); uncontrolled metabolic disorders; chronic use of anti-inflammatory, immune-suppressive, or bone/mucosa-affecting drugs; patients with untreated periodontal conditions; alcohol and drug abuse; pregnancy or lactation; or those unable to provide consent.

### 2.3. Implant procedure

After implant assessment and subsequent recruitment into the study, a standard work-up and preparation for implant placement was performed. The choice of type of dental implant (tapered or parallel, internal or external connection, implant length, implant width) was made based on clinical and restorative indication, independent of the study. All implants placed in this study were Zimmer Biomet® type implants (Zimmer Biomet Dental, FL, USA) with an Osseotite® surface technology. Second stage implant surgeries were all performed after a standard healing period of 3 months.

### 2.4. Implant stability measurements

Two operators (IR and IP) collected all implant stability measurements by utilising the Osstell® and Periotest™ devices.

#### 2.4.1. Osstell® ISQ device

The measurements for the Osstell® ISQ device were recorded after attaching the appropriate smartpeg to the implant and placing the probe tip of the Osstell® device close to the head of the smartpeg. Smartpegs were attached to the implant by tightening with finger pressure. Two measurements were taken from a mesio-distal and bucco-lingual direction, and an average for each was derived. A mean (overall) ISQ based on the 4 measurements was also calculated. These readings were recorded as the implant stability quotient (ISQ). At T3, restorative supra-structures were removed, and a smartpeg was placed again for measurements of ISQ values consistent with methodology at T1 and T2.

#### 2.4.2. Periotest™ device

Measurements for the Periotest™ device were recorded with the metal “slug” tapped against the surface of a healing abutment connected to the dental implant and about 3 mm coronal to the implant head. The head of the tapping pistol was directed in a perpendicular direction towards the healing abutment and at a distance of about 2 mm. Two readings were recorded per implant fixture. For analysis the average of these two readings was taken as the “Periotest value” (PTV). At T3, restorative supra-structures were removed, and a healing abutment was placed again for measurements of PTV values consistent with methodology at T1 and T2.

### 2.5. Other variables

Baseline data collection included the following demographic factors; age, gender and smoking history. Clinical measurements were categorized into implant position, site, surgery and fixture characteristics. Implant position was recorded by jaw. Implant site factors included bone thickness, presence or absence of an adjacent tooth both mesially and distally, and simultaneous augmentation of the surgical site. At T3 peri-implant soft tissue status was recorded as well as marginal bone levels.

### 2.6. Statistical analysis

An *a priori* power analysis was carried out. Based on the available evidence that has previously evaluated the correlation between the Osstell® and Periotest® devices, a power calculation was performed to estimate the sample size required to achieve power for the statistical analysis. This determination ensures a 95% chance of rejecting the null hypothesis when the projected population effect size is 0.6 and the alpha level for the test is 0.05. Based on this arithmetic 30 implants were required.

The unit of measurement was taken as the implant (rather than patient). Implant stability measurement data were not normally distributed (Kolmogorov-Smirnov test). Correlation testing between the Osstell® and Periotest**^™^** devices was therefore carried out utilising Spearman's rank correlation for each time point. Analysis of the difference between clinical factors and stability measurements was compared *via* the Wilcoxon rank-sum test. Implant stability measurements by time points were compared using a Kruskal-Wallis test. The level of statistical significance was set at *p *< 0.05. Analyses were performed using Stata 15 (StataCorp. 2017. Stata Statistical Software: Release 15. (College Station, TX: StataCorp LLC.).

## 3. Results

As part of this prospective cohort study 29 subjects were consecutively recruited by convenience sampling and data was collected for a baseline (T1) *n* = 68 dental implants. At second stage surgery (T 2), 29 patients provided data on *n* = 67 implants (one patient didn't attend his appointment). At 3 years post implant placement (Time point 3), 25 patients provided data on *n* = 58 implants. One implant failure was experienced by a patient that contributed to 2 implants in this study, and 4 patients (9 implants) did not attend their 3-year review appointment).

The mean age of subjects at baseline (T1) was 42.5 years with a range of 55 years from a minimum of 19 up to 74 years of age. There were 16 female (55%) and 13 male participants (45%). 10% of the participants were classified as current smokers, 14% were former smokers and 76% never smoked. From a general health point of view, no participants had a history of bisphosphonate use. Forty-one of the dental implants were placed in the maxilla representing 60% of cases. Fifty-five implants were placed in the anterior region representing 81% of cases.

In Table 1 the median ISQ in the bucco-lingual direction, mesio-distal direction and median value for both directions for all time points can be seen. The median ISQ value was 73.25 at implant placement, decreased to 72.75 by second stage surgery and increased to 74 at 3 years from placement. In the same table, Periotest™ values demonstrated a progressive decrease in PT value from −4 to −5.5 to −6 and demonstrated an increase in stability from implant placement to fit of prosthesis. Included were also boxplots depicting the median, 25th and 75th percentiles (inter-quartile range), and the minimum and maximum values (range) for implant stability quotient and Periotest**^™^** values obtained at T1, T2 and T3. There was a statistically significant difference across Periotest values observed at T1, T2 and T3, (*p < 0.001)*. No statistically significant differences were observed across the ISQ values at different time points (Table 1).

**Table 1:** Implant stability measurements by time point.

	T1 (Implant insertion)	T2 (Second stage surgery)	T3 (3 years post insertion)	*p* ^a^
**Buccal Lingual ISQ,**				
median (IQR)	73.5 (67–75)	73 (66–76)	75 (70–78)	0.12
range	30 to 89	35 to 89	60 to 85	
**Medial Distal ISQ,**				
median (IQR)	74 (66–76)	73.5 (68–76)	74 (71–77)	0.32
range	48 to 89	42 to 90	58 to 85	
**Mean ISQ,**				
median (IQR)	73.25 (67–75)	72.75 (67–76.5)	74 (70.5–77)	0.2
range	39-89	42-85.5	59-85	
**PTV value,**				
median (IQR)	−4 (−6, −2)	−5.5 (−7, −3)	−6 (−7, −5)	<0.001
range	−8 to 29	−8 to 3	−8 to −3	

The range of ISQ values obtained, which can also be described as the difference between the lowest and highest ISQ value recorded, was shown to decrease from T1 to T3; this was observed with ISQ values recorded in the bucco-lingual and mesio-distal direction and as a mean of ISQ values in both orientations. Overall the minimum ISQ value recorded in the study was 30 and the maximum was 89. From T1 to T3 in the bucco-lingual direction the range of ISQ values narrowed from 59 ISQ points at T1 to 25 at T3, in the mesio-distal direction the ISQ values narrowed from 41 at T1 to 30 at T3 (Table 1).

A weak to moderate level of statistically significant correlation was observed across time points for ISQ and Periotest™ values. The strongest relationship was demonstrated between the ISQ and the PT values at T2 with a coefficient of −0.35 (*p < 0.01*) (Table 2).

**Table 2:** Spearman's rank correlations for the various osstell® and periotest® measurements at each time point, *n *= 58.

	T1 (Implant insertion)		T2 (Second stage surgery)		T3 (3 years post insertion)	
	rho	*p*	rho	*p*	rho	*p*
Bucco-lingual ISQ vs. PTV	−0.16	0.23	−0.29	0.03	−0.24	.06
Mesio-distal ISQ vs. PTV	−0.34	<0.01	−0.35	<0.01	−0.26	0.05
Mean ISQ vs. PTV	−0.26	0.05	−0.35	<0.01	−0.28	0.04

Clinical characteristics were evaluated and compared to mean implant stability measurements of the Osstell® and Periotest™ devices at T1 and T3 as well as with the differences in the values from T1 to T3 (Table 3). In general, Periotest™ seemed to be more sensitive to local conditions as it identified differences in implant stability between maxilla and mandible (T1 and T1–T3), as well as between implants that demonstrated MBL or not at year 3 (T1–T3).

**Table 3:** Wilcoxon rank-sum for osstell® and periotest® measurements by clinical feature at T1, T3, and difference T1 to T3, *n *= 58.

Clinical feature	T1 (Implant insertion)				T3 (3 years post insertion)				Difference (T1 to T3)			
	Ostell		Periotest		Ostell		Periotest		Ostell		Periotest	
	z	*p*	z	*p*	z	*p*	z	*p*	z	*p*	z	*p*
Jaw	−1.85	0.06	**4** **.** **03**	**<0** **.** **001**	−0.83	0.4	1.3	0.19	1.13	0.26	**−3** **.** **11**	**<0** **.** **01**
Maxilla (*n *= 36)												
Mandible (n-22)												
Buccal bone thickness	−1.79	0.07	**2** **.** **64**	**<0** **.** **01**	0.66	0.51	1.77	0.08	**2** **.** **75**	**<0** **.** **01**	−1.01	0.31
<2 mm (*n*=27)												
≥2 mm (*n *= 31)												
Lingual bone thickness	0.15	0.88	1.4	0.16	1.67	0.1	1.7	0.09	1.03	0.3	0.01	0.99
<2 mm (*n *= 28)												
≥2 mm (*n *= 30)												
Adjacent tooth	0.85	0.39	0.05	0.96	1.72	0.09	−0.87	0.38	−0.06	0.95	−0.04	0.97
Present (*n *= 32)												
Not present (*n *= 26)												
Simultaneous Grafting	0.5	0.62	−1.51	0.13	−0.02	0.98	−0.68	0.5	−0.69	0.49	1.18	0.24
Yes (*n *= 11)												
No (*n *= 47)												
Current or former smoker	−0.77	0.44	**−2** **.** **14**	**0** **.** **03**	1.68	0.09	**−2** **.** **31**	**0** **.** **02**	1.88	0.06	1	0.31
Yes (*n *= 13)												
No (*n *= 45)												
Presence of plaque year 3	−0.06	0.96	−0.84	0.4	0.38	0.7	−1.77	0.08	0.32	0.75	−1.19	0.23
Yes (*n *= 26)												
No (*n *= 32)												
Peri-mucositis year 3	1.61	0.11	−0.46	0.65	1.99	0.05	0.27	0.79	0.11	0.91	0.93	0.35
Yes (*n *= 13)												
No (*n*-45)												
Peri-implantitis year 3	−0.26	0.8	2	0.05	**2** **.** **24**	**0** **.** **03**	−1	0.32	**2** **.** **2**	**0** **.** **03**	**−2** **.** **17**	**0** **.** **03**
Yes (*n *= 2)												
No (*n *= 56)												
Marginal bone loss year 3	0.18	0.86	1.27	0.2	1.11	0.27	−1.8	0.07	0.41	0.68	**−2** **.** **16**	**0** **.** **03**
Yes (*n *= 6)												
No (*n *= 52)												

## 4. Discussion

Much of the historical research published on this topic has recorded stability measurements at weekly intervals commencing with implant placement (13, 14). The aim of those studies was to closely scrutinise the alterations in implant stability that occur during osseointegration. In contrast, our study sought to elucidate knowledge that may be more directly applied to clinical practice. A statistically significant increase in implant stability was recorded by the Periotest™ device over time. From implant placement to fit of prosthesis, the median PT value depreciated from −4 to −6 and narrowed in range of values from T1 to T3. The PT value ranged from 29 to −8 at insertion and −3 to −8 three years later.

Early research on the Periotest™ by Truhlar and colleagues assessed implant stability at second stage surgery on 1,838 root form implants (15). This investigation sought to establish normative ranges for the Periotest™ and to correlate the device with various bone densities. For stable implants at second stage surgery the mean Periotest value was −3.37 +/− 3.25. The study identified the influence of bone quality on PTVs with implants inserted in dense cortical bone displaying a lower mean PTV of −3.82 +/−3.04 in contrast to implants placed in softer trabecular Type IV bone having a mean PTV of −1.29 +/−3.57 (15). Further studies confirmed these findings identifying an average PTV of −3.5 in their study that evaluated the stability of the bone implant complex over 60 months with the Periotest™ machine (16). Early research by Teerlinck and co-workers applying the Periotest™ to dental implants found a range of Periotest values between −4 and +2 (17). A more recent study from 2012 that evaluated the relationship between implant stability and bone quality with the Periotest™ device identified an average range of −5 to +5 for PTVs (10). We could postulate that these differences may be due to the implant system used or the gradual improvement of the implant surfaces over the last 20 years. As improved surfaces allowed for better ossointegration, the stability values improved. This potential for difference between implant stability values and implant systems has been previously referenced in the literature, specifically for resonance frequency analysis (RFA) devices (2).

A different trend was identified for the Osstell® device with a non-significant increase in mean RFA values identified over time. A median ISQ value of 73.25 was determined at implant placement, decreasing to 72.75 at second stage surgery and reaching the median value of 74 three years following placement. This doesn’t equate to the body of evidence available. However the RFA device did demonstrate a narrowing of values recorded from T1 to T3, 39–89 ISQ at implant placement and 59–85 ISQ three years following placement, which does correspond to the published studies. Past research has suggested that a merging of high and low stability measurements to a narrower normalized range does occur over time and this process reflects the density of bone that the implant was placed into and the mechanism of osseointegration (18, 19). A normative range of 61–85 ISQ was identified in the study herein for Osseotite® Zimmer Biomet® implants while in previous articles a normative range of 57–70 ISQ has been proposed for Straumann® fixtures (13), and 57–82 for Branemark® implants (19).

The results of this study demonstrated a weak/moderate level of correlation between the Osstell® & Periotest™ measurements across all time points. There was a moderate level of correlation between the stability values recorded with RFA and Periotest instruments at second-stage surgery. This degree of correlation was similar for mesio-distal and mean ISQ values compared to PTV values but lower between Bucco-lingual and PTV values. Weak to moderate correlation was also identified across all time points when the mean ISQ value was paired with the Periotest values. This correlation was weaker at T1 (*r* = −0.26, *p = 0.05*), higher at T2 (*r* = −0.35, *p < 0.01*) and lower again at T3 (*r* = −0.28, *p = 0.04*). This result is consistent, albeit with a weaker correlation, than those of Merhab and co-workers (*r* = −0.52*, p < 0.001*), and Zix and team (*r = *−*0.650*) (20, 21). Both of those studies demonstrated a moderate correlation between ISQ and PTV values at implant placement and loading. Their larger sample size and use of a different implant system may explain the stronger correlation observed in these studies. Additionally, the correlations in the study herein were weaker when compared with work by Seong and team as well as a series of studies by Lachmann and team (22–24). They identified a correlation of (*r* = −0.852), (*R*^2 ^= 0.8*, p < 0.0001)* and (*R*^2 ^= 0.89*, P < 0.0001)* respectively*.* These investigators performed laboratory based research on the devices in contrast to the clinical nature of our study. The easier access, direction and orientation when operating these machines in a non-clinical environment has been proposed as a reason for the higher level of correlation observed in an experimental study setting.

Another aspect to consider when comparing RFA and Periotest™ devices is the requirement for two measurements with the Osstell® machine in contrast to one reading with the Periotest™ machine. The importance of recording a bucco-lingual measurement and separate mesio-distal measurement with the Osstell™ is well documented through manufacturer guidance and in the literature, however most studies have only analysed the mean of the ISQ value recorded in both directions. This limited evaluation of the true functionality of the Osstell® may hide superiority of this device in comparison to the Periotest™ machine. From a clinical perspective bone deficiencies in the alveolar ridge are most prevalent in the bucco-lingual dimension rather than the mesio-distal. Single Periotest™ measurements may fail to reveal differences in implant stability as a direct relationship to bony deficiency in the bucco or lingual region. The findings of this study, however, do not seem to support these interpretations.

Statistically significant higher ISQ values were recorded from implants placed in the lower jaw when compared to the ISQ values recorded from implants placed in the upper jaw. Similarly, lower PTV values were recorded in the mandible compared to the maxilla. These findings are in agreement with several other published articles in which higher RFA values and lower Periotest values were recorded in the mandible (19, 25). Research by Seong et al. specifically utilised Biomet 3i® dental implants and evaluated the relationship between stability measurements and jaw position (24). In agreement with the study herein, they reported significantly different mean stability values for the maxilla and mandible when measured by the Osstell® and the Periotest™ devices.

Intriguingly, from our study, the two dental implants that demonstrated the lowest stability values were from augmented sites. One of the surgical areas received guided bone regeneration prior to implant placement and the other was simultaneously augmented at the time of implant placement. During the course of the study the implant placed in the previously grafted site was later reported as a failure. A bucco-lingual ISQ value of 41 and mesio-distal ISQ value of 57 (Mean ISQ 49) was recorded several weeks prior to the clinical failure of the dental implant. These values were recorded at the time of implant exposure and in this period there were no associated clinical or radiographic signs that indicated future implant failure. The initial ISQ values at implant placement were 68 and 69 for bucco-lingual and mesio-distal direction respectively. The Periotest values demonstrated a similar trend with an initial value of −6 at implant insertion and an increased value of 3 at second stage surgery. These changes in stability measurement values are reflective of reduced implant stability and support the argument that these devices may act as a prognostic indicator for implant failure. The measurement of a significantly reduced ISQ value in the absence of negative symptoms or implant mobility are consistent with the literature from Friberg et al., in which an implant failed several weeks after a significantly reduced ISQ value had been recorded despite the absence of any other negative clinical signs that would indicate potential future implant failure (26). Interestingly a study produced by Nedir and team proposed a cut-off ISQ value that would act as a predictor for implant stability. Based on the results of their study they proposed an ISQ of 47 and this yielded a sensitivity of 100%. The findings of the implant failure in our case correspond quite well with those of Nedir and colleagues (14). Similarly, research by Noguerol and co-workers suggested a cut-off point of −2 for the Periotest™ as a prognostic indicator for implant loss (27). These results are approximate with those of our study and support the proposition that the RFA and Periotest™ devices may provide clinical value as prognostic indicators for implant failure. Additionally, based on the results from the study herein RFA and PTV measurements seem to be useful tools for identifying not only failing osseointegration but also marginal bone loss (MBL) around implants with established peri-implantits. As a result, using these devices as adjuncts to clinical examination can help in diagnosing peri-implant diseases earlier and more efficiently.

The application of a clinical study model is a definite advantage to this study in contrast to much of the published evidence based on experimental and pre-clinical research. The use of a single implant system is an obvious strength of this study and to the best of the authors knowledge this is the first study of this type that has utilised Zimmer Biomet® oral implants. The pooling of a number of implants in a small number of patients is an obvious limitation of the study and increases risk of bias particularly when analysing clinical factors. Ideally single implants in individual participants would be a more favourable format for analyses of this type. In our study hand tightening of smartpegs was performed and this is supported by some of the literature (28) however other studies dispute this recommendation. They suggest that a specific controlled force should be applied to tighten the smartpeg to the implant to ensure accurate readings (29, 30). The majority of studies that investigate RFA measurement devices have manually tightened the smartpegs and there is nominal reference to the use of controlled force. In the clinical environment it would be reasonable to assume that hand tightening of the smartpeg is the standard practice.

This study has incrementally added to our understanding of implant stability measurement devices in particular the Osstell® and Periotest™. The findings of correlation between implant stability measurements and interrelation to implant position have strengthened and confirmed the consistency of previous publications. This paper has proposed a normative range for ISQ and PT values for Zimmer Biomet® implants and supports the suggestion that a narrowing of stability measurement values develops from implant placement to fit of prosthesis. Finally, this research has strengthened the proposal for these devices to act as prognostic indicators for implant failure. Overall, the study design employed in our research addressed several limitations of previous studies. Future research should however aim to resolve the numerous methodological deficiencies outlined above to enhance the quality of evidence in this field.

## 5. Conclusion

Based on the results of this study there was a weak to moderate level of correlation between values recorded between the two measurement devices at implant placement, implant exposure and three years following placement. For both the Osstell® and Periotest™ a narrowing of the range of stability values was observed from T1 to T3. In general, Periotest™ seemed to be slightly more sensitive in highlighting differences in measurements affected by local conditions and using them as adjuncts to clinical examination seemed to be useful in establishing an early diagnosis of either loss of osseointegration or peri-implant diseases.

## Data Availability

The raw data supporting the conclusions of this article will be made available by the authors, without undue reservation.
